# Case Report: A 69-year-old woman with dermatopathic lymphadenopathy and hypercalcemia after COVID-19 infection

**DOI:** 10.3389/fimmu.2026.1702218

**Published:** 2026-03-04

**Authors:** Danli Hu, Yanchun Li, Shen Shen

**Affiliations:** 1 Department of General Medicine, Beijing Chao-yang Hospital, Capital Medical University, Beijing, China; 2 Department of Nephrology, Beijing Chao-yang Hospital, Capital Medical University, Beijing, China

**Keywords:** COVID-19 infection, dermatopathic lymphadenopathy, hypercalcemia, immune dysfunction, inflammation

## Abstract

**Patient concerns:**

A 69-year-old female presented with hypercalcemia, recurrent fever, and lymphadenectasis following COVID-19 infection. Positron emission tomography/computed tomography (PET-CT) revealed no suspicious malignancy. Lymph node biopsy from bilateral inguinal areas suggested Langerhans cell histiocytosis, supporting a possible diagnosis of dermatopathic lymphadenitis or rare Langerhans cell histiocytosis. The immunohistology staining showed that the proliferated Langerhans cells in lymph nodes expressed CD68, CD1a, and S100, further supporting the diagnosis of dermatopathic lymphadenitis.

**Diagnosis and intervention:**

The patient was diagnosed with dermatopathic lymphadenopathy. The patient underwent a therapy of prednisone acetate at 30 mg per day. Then, glucocorticoid was gradually decreased by 2.5 mg per 2 weeks, until it reached 7.5 mg per day. Salmon calcitonin was subcutaneously injected to decrease the calcium level and relieve pain during hospitalization.

**Outcomes:**

During the 3 months of treatment, the systemic symptoms were significantly alleviated. The lymph nodes of the right inguinal area and the serum calcium level decreased to normal. She remained in remission for an additional 4 years.

**Lessons:**

Coexistence of the two unrelated diseases, dermatopathic lymphadenopathy and hypercalcemia, was rare. Immune dysregulation and persistent inflammatory responses post-COVID-19 infection may be the potential mechanism for hypercalcemia.

## Presentation

A 69-year-old female patient was admitted to the hospital with a 6-month history of recurrent fever and generalized aches and pains as well as a 6-day history of hypercalcemia.

The patient had a COVID-19 infection before April 2023, presenting with fever and green sputum. She went to a local clinic and received an intravenous treatment of cephalosporin and non-steroidal anti-infective medicine for 3 days. Following the intravenous therapy, she developed a generalized edema of the lower limbs and fatigue. Then, she was immediately admitted to the pneumology department of a tertiary hospital and underwent chest computed tomography examinations. The scan revealed bilateral lower lobe pneumonia and bronchiectasia. She received a treatment of cefperazone-sulbactam and diuretics for 2 weeks. Then, her condition showed improvement, and she was discharged. Her blood calcium level was normal during hospitalization.

After discharge, the patient still had a recurrent low-grade fever and general aches for 4 months. On August 2, 2023, she underwent a blood test, and the laboratory testing indicated the following: leukocyte count of 11.2 × 10^9^/L (3.5-9.5 × 10^9^/L), hemoglobin of 7.9 g/dL (11.5–15 g/dL), hematocrit of 22.6% (35-45), platelet count of 280 × 10^9^/L (125-350 × 10^9^/L), eosinophil count of 1.96 × 10^9^/L(0.02-0.52 × 10^9^/L), albumin of 29 g/L, creatinine level of 153 umol/L (44–97 umol/L), and estimated glomerular filtration rate of 30 mL/min per 1.73 m^2^), which was increased from her baseline of 101 umol/L, and serum calcium level of 3.56 mmol/L (reference 2.0-2.25 mmol/L) and alkaline phosphatase level of 649.0 U/L (50-1 35U/L). Her urinalysis revealed negative results for urine protein and red blood cells. Her total cholesterol was 1.21 mmol/L, triglyceride was 1.21 mmol/L, and total parathyroid hormone (PTH) was 8.8 pg/mL (reference 18.5-88.0). Serum 25-hydroxyvitamin D3 was 17.1 ng/mL (reference 30–100 ng/mL). She presented to our hospital and was diagnosed with renal insufficiency and anemia. She was treated with erythropoietin (EPO) before being admitted to the hospital. Throughout the course of the condition, the patient reported intermittent fever and consistent bone pain. She had no episodes of skin rash, photosensitivity, polyarthralgia, or significant hair loss. The patient denied symptoms such as dry mouth, dry eyes, chunk-like tooth loss, hemoptysis, abdominal pain, and black stools. Her appetite decreased since the onset of the disease. Her sleep, stool, and nocturia were normal. She experienced a weight loss of 4 kg over the previous 6 months.

For the past 2 years, a chronic pruritic rash on the calves had occurred intermittently after daily exercise. Six months ago, she had a cystic lesion of the pancreas and then was diagnosed with intraductal papillary mucinous neoplasm of the pancreas after PET-CT examination. The SUVmax was 8.5 in mediastinal and bilateral hilar lymph nodes, suggesting bronchiectasis with infection. Four months ago, the patient was diagnosed with coronary heart disease and a previous myocardial infarction. She was also diagnosed with intermuscular deep vein thrombosis in both calves, and she received rivaroxaban for anticoagulation. Three days ago, she had a diagnosis of malnutrition and been treated with enteral nutritional powder.

Family history: Her father died of gastric cancer, and her mother died of colorectal cancer. Her elder brother had bone cancer, and her younger brother had early-stage colorectal cancer. Her three sisters suffered lung cancer, concurrent cerebral hemorrhage, and bladder cancer, respectively. She did not drink and smoke.

## Assessment

On admission, the patient had a fever of 38.3 °C, and her blood pressure was 127/74 mmHg, with no orthostatic drop. She was thin but not cachectic. She had bilateral multiple enlarged supraclavicular, axillae, and inguinal lymph nodes that were moveable, moderately firm, and non-tender. Mild edema is noted in both lower limbs. She had no abdominal tenderness, had normal bowel sounds, no palpable masses, no rebound tenderness, and no noticeable distention. Her cardiac, spleen, and pulmonary examinations were unremarkable.

Laboratory testing indicated a white cell count of 13.9 × 10^9^/L, hemoglobin of 7.4 g/dL (11.5–15 g/dL), platelet count of 171 × 10^9^/L (125-350 × 10^9^/L), and eosinophil percentage of 28.5%. A comprehensive metabolic panel showed a calcium level of 3.56 mmol/L (2.11-2.52 mmol/L), magnesium level of 0.61 umol/L (0.65-1.25 umol/L), and increased alkaline phosphatase level of 649 U/L (50–135 U/L). Serum PTH was 8.8 pg/mL (15–65 pg/mL). The amylase and lipase values and that of lactate dehydrogenase were within normal range. The autoantibody results, including ANA and ANCA, were negative.

Superficial lymph node ultrasonography showed bilateral enlarged inguinal lymph nodes, approximately 1.7 cm × 1.0 cm on the left and 1.9 cm × 0.8 cm on the right. The multiple enlarged supraclavicular and axillae lymph nodes were visible on ultrasonography, the largest of which was 2.7 cm × 0.9 cm on the left. The color Doppler ultrasound found intermuscular venous thrombosis of the lower extremity. The urinary ultrasound disclosed a bilateral medullary sponge kidney. Lung ventilation-perfusion scan revealed reduced blood flow perfusion in the medial segment of the right middle lobe and posterior apical segment of the left upper lobe, indicating a high likelihood of pulmonary embolism. Additionally, there were multiple non-segmental ventilation-perfusion matching changes in both lungs, suggestive of parenchymal lung disease. Renal ultrasound showed abnormal echogenicity in the renal medulla, raising the possibility of sponge kidney. A further bone scan indicated unevenly increased tracer uptake in the skull, bilateral clavicles, bilateral humeri, bilateral radii, ulnae, and bilateral femurs.

**Figure 1:**
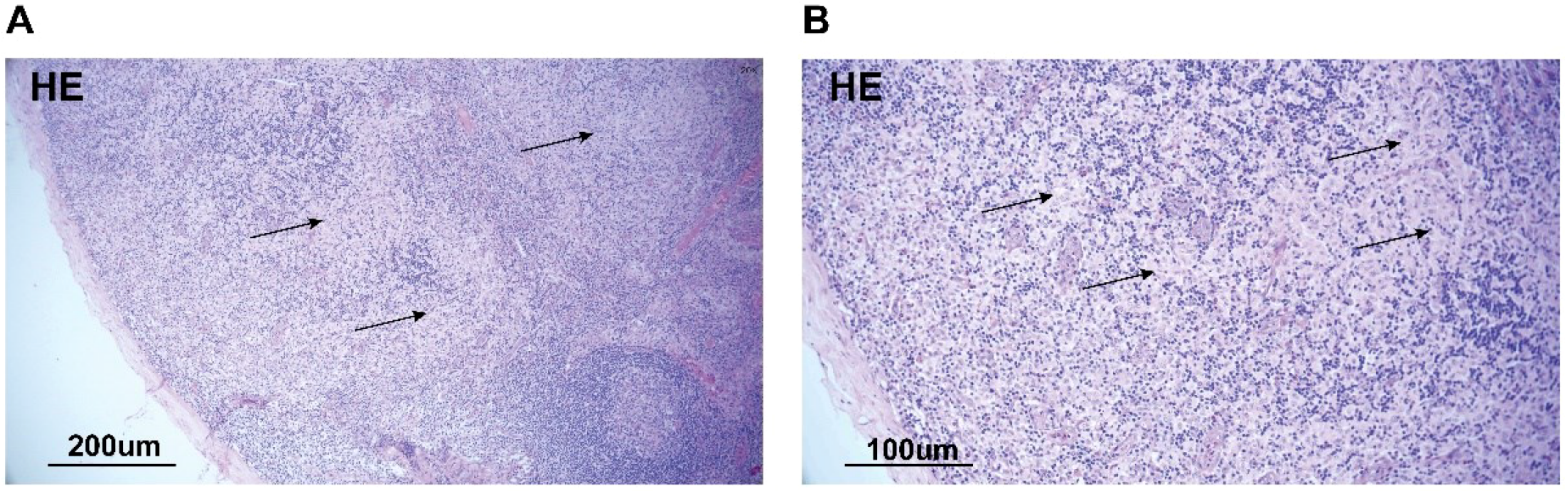
HE staining of inguinal lymph node. Biopsy specimen of inguinal lymph node. Hematoxylin and eosin staining of the biopsy specimen is shown at low magnification **(A)** and at high magnification **(B)**. The specimen shows extensive necrosis with some inflammatory cells, with no evidence of lymphoma or other cancers.

**Figure 2:**
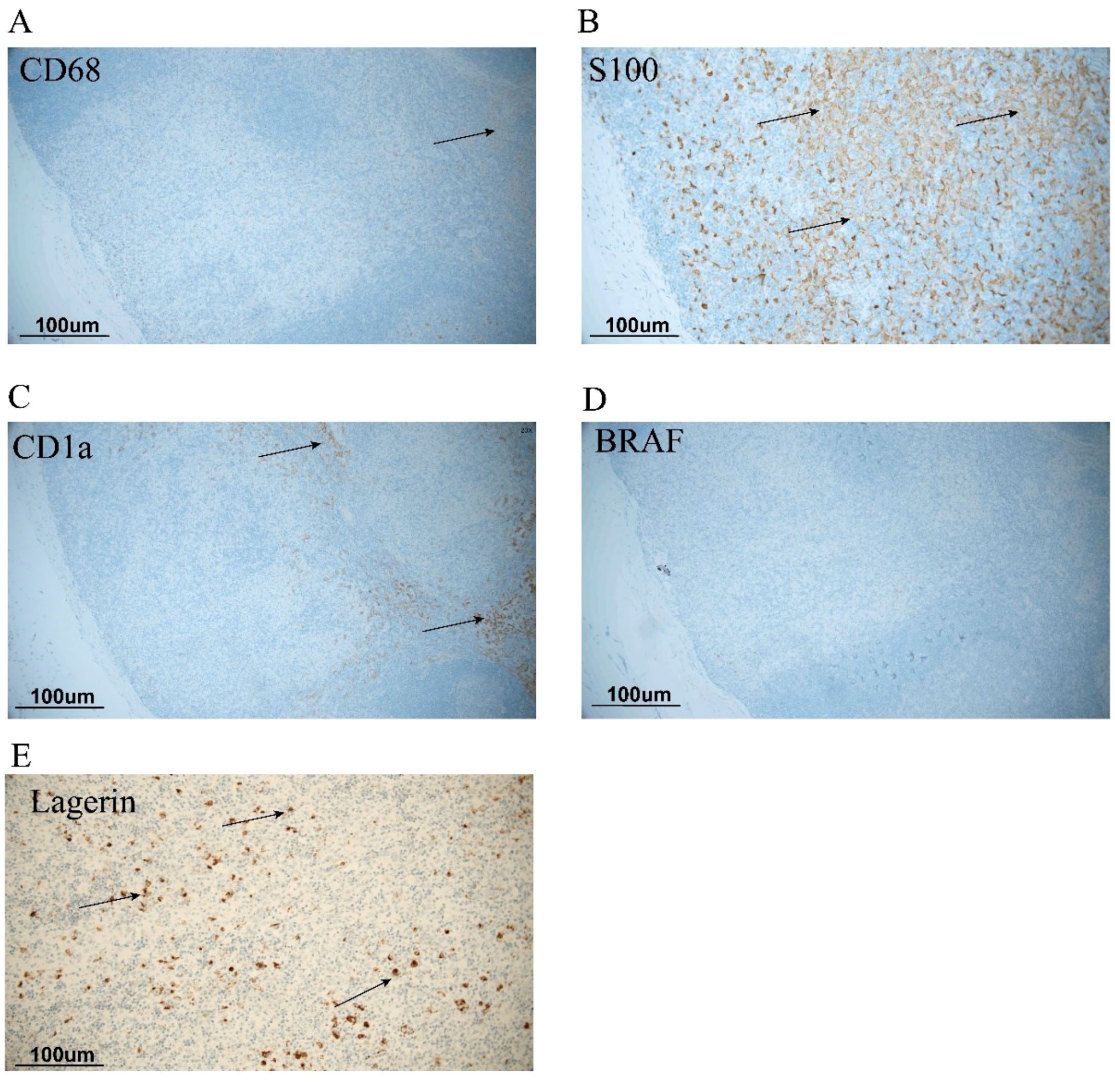
Immunohistochemical staining of CD68, S100, and CD1a. Immunohistochemical (IHC) staining of CD68, S100, CD1a, BRAF, and Lagerin. **(A)** IHC staining of CD68. **(B)** IHC of S100. **(C)** IHC of CD1a. **(D)** IHC of BRAF V600E. **(E)** IHC of Lagerin. The positive staining indicated the activated Langerhans cells. S100 and CD1a double-positive cells suggested dermatopathic lymphadenitis. The arrow indicates positive Langerhans cells.

A subsequent bone marrow biopsy showed a granulocyte count of 66.5%, among which the proportion of the eosinophils was 21%. To further identify the cause, we performed inguinal lymph node biopsy. HE staining did not find atypical cells (**Figure 1**). Immunohistochemical staining revealed positive staining for CD68, CD1a, and S100. BRAF gene testing of blood yielded negative results (**Figure 2**).

## Discussion

The patient experienced intermittent low-grade fever in the afternoon, generalized lymphadenopathy, pruritus, and widespread body pain. Laboratory findings demonstrated recurrent hypercalcemia, elevated eosinophil count, decreased parathyroid hormone (PTH) levels, and negative tumor biomarkers. To certify the diagnosis, we considered the likely causes of hypercalcemia, including parasitic infections, primary hyperparathyroidism, sarcoidosis, hematological tumors, and solid tumors.

The likely cause of hypercalcemia with a suppressed PTH was a malignancy. These solid tumors often secreted parathyroid-related peptide (PTHrP), which increased the serum calcium level by stimulating PTH receptors, leading to osteolysis. The resultant hypercalcemia caused an appropriately suppressed PTH level by means of feedback mechanisms (1). Considering the malignancy family history and her signs, the patient was at a high risk of cancer. However, the tumor biomarkers, including AFP and CEA, were negative. The positron emission tomography-computed tomography (PET-CT) ruled out solid tumor lesions.

The patient’s case was highly suspicious of lymphoma, which often caused elevated PTHrP or 1,25-dihydroxyvitamin D3 levels (2). In our case, immune cell typing in peripheral blood through flow cytometry was also negative. The lymphoid and marrow biopsy did not find evidence of heterogeneous tumor cell and necrosis. Serum and urine electrophoresis and immunofixation electrophoresis did not suggest abnormal monoclonal proteins or elevated light chains. Therefore, we did not consider the diagnosis of lymphoma, myeloma, and other blood system lesions.

The patient also needed to be differentiated from Langerhans cell histiocytosis. The immunohistology results showed S100 and CD1a positivity in the nuclei/cytoplasm of the proliferated Langerhans cells, which was a more specific marker of dermatopathic lymphadenitis (3, 4). Moreover, the immunohistology results revealed negativity in BRAF. This could further rule out Langerhans cell histiocytosis. Our provided H&E images illustrated the preserved lymph node architecture and the non-destructive, interstitial pattern of involvement, which was classic for a reactive process like DL.

Sarcoidosis also needed to be excluded. HE staining did not find granulomatous structures in this case. The chest CT did not reveal lymph node lesions at the bilateral pulmonary hilum or grid-like pulmonary infiltrates, which could also exclude the diagnosis of granulomatous disease.

DL was a rare benign lymphatic hyperplasia commonly associated with exfoliative or eczematoid dermatitis (5–7). However, DL accompanied by hypercalcemia post-COVID-19 was rare. pDC infiltration was at significantly higher levels in various pathological tissues, such as the skin of patients with systemic lupus erythematosus and in the muscle tissue of patients with idiopathic inflammatory myopathies and COVID-19 infection. The DCs in the pathologic condition had increased migration ability and contributed to the secretion of myeloid growth factors, including RANKL, TNF-α, and IL-6, and induced JAK2-STAT5 signaling pathway and RANKL/OPG signaling (8). The IL-6-mediated osteoclast activation may cause bone reabsorption and hypercalcemia.

Other considerations such as parasitic infection were also ruled out because there was no evidence of infection in imaging and biopsy. Another often-overlooked cause was primary hyperparathyroidism (9). Though the patient had abnormal alkaline phosphatase and osteoporosis, the suppressed PTH level and parathyroid ultrasound ruled out the consideration.

Considering the diagnosis of dermatopahic lymphadenitis, the patient underwent a therapy of prednisone acetate at 30 mg per day, according to her weight. The glucocorticoid dose was gradually tapered from an initial dose of 30 mg daily down to 7.5 mg daily, which was decreased by 2.5 mg every 2 weeks. Salmon calcitonin was subcutaneously injected to decrease the calcium level during hospitalization. Her overall pain was significantly alleviated, and the fever resolved. During follow-up after 1 month, her inguinal lymph node was reduced. Following 3 months of treatment, she remained in remission for an additional 4 years.

The case representation had several limitations. First, we lack direct dermatological documentation such as photographs, specialist consultation, or biopsy because we focus on managing life-threatening conditions. Second, we lack data on the measurement of cytokines, including IL-6, TNF-α, RANKL, PTHrP, and 1,25-dihydroxyvitamin D. We speculate that the cause of hypercalcemia may be the cytokine-mediated osteoclast activation, but we have no definitive proof.

We first report a rare case with coexistence of DL and hypercalcemia post-COVID-19. Immune dysregulation and persistent inflammatory responses post-COVID-19 infection may be the potential mechanism for hypercalcemia.

## Data Availability

The raw data supporting the conclusions of this article will be made available by the authors, without undue reservation.
